# General Health, Systemic Diseases and Oral Status in Adult Patients with Coeliac Disease

**DOI:** 10.3390/nu12123836

**Published:** 2020-12-15

**Authors:** Alessandro Nota, Silvio Abati, Floriana Bosco, Isabella Rota, Elisabetta Polizzi, Simona Tecco

**Affiliations:** Dental School, Vita-Salute San Raffaele University and IRCCS San Raffaele Hospital, 20132 Milano, Italy; nota.alessandro@hsr.it (A.N.); abati.silvio@hsr.it (S.A.); boscofloriana@gmail.com (F.B.); rota.isabella@hsr.it (I.R.); polizzi.elisabetta@hsr.it (E.P.)

**Keywords:** coeliac disease, oral diseases, oral prevention, gingival bleeding, sleep-related breathing disorders, oral health, enamel defects, interceptive orthodontics

## Abstract

The prevalence of coeliac disease in the general population is 0.5–1%; however, most patients remain undiagnosed until adult age. In some cases, the onset is represented by sub-clinical signs, some of which can be found in the mouth. The aim of this research was to identify any associations between the clinical characteristics of coeliac disease and oral manifestations. A structured questionnaire was administered to a group of 237 individuals with coeliac disease. 100% of the subjects fully completed the questionnaire. Among them, 182 (76.7%) were female, 64 patients (27%) were aged 15 to 24 years, 159 (67%) were aged 25 to 55 and 14 (6%) were aged 56 and over. Significant associations were observed in caries prevalence and dentin sensitivity; in addition, an inappropriate diet was related to oral manifestations; following a gluten-free diet could be important to control the gingival bleeding levels and to manage oral symptoms associated to coeliac disease. In general, the presence of inflammatory symptoms in the mouth seems to be associated with general symptoms of inflammation related to coeliac disease.

## 1. Introduction

Coeliac disease is an immune-mediated disease, typical of genetically predisposed individuals; it is caused by gluten [1]. A gluten-free diet is the only treatment strategy accepted in these patients [2]. The prevalence of coeliac disease in the general population is 0.5–1%; however, most patients remain undiagnosed until adult age. There are several clinical onsets of the disease, the most common caused by malabsorption (iron deficiency anemia, hypovitaminosis etc.) [3]; in some cases, the onset is represented by sub-clinical signs, some of which are found in the mouth (herpes-like lesions, recurrent aphthous stomatitis, hypoplasia and dyschromia of the enamel, etc.) [4]. It is characterized by a variety of symptoms, both intestinal and extra-intestinal, including oral manifestations [5]. Coeliac disease can develop at any age after people start consuming food that contains gluten. If untreated, coeliac condition can lead to a worsening of health, for example the development of other autoimmune disorders, such as type I diabetes, multiple sclerosis, dermatitis herpetiformis, anemia, osteoporosis, infertility and miscarriage, neurological conditions such as epilepsy and migraine, short stature and intestinal tumors [6]. The aim of this research is to identify, through the analysis of a questionnaire distributed to coeliac adult patients, any association between the clinical systemic characteristics of coeliac disease and oral manifestations, with the ultimate purpose of improving the clinical dental management of this category of patients. The primary outcome was the association of the clinical systemic signs and symptoms associated with coeliac disease with the lifestyle of patients with intra-oral manifestations.

## 2. Subjects and Methods

This is an observational study based on the data derived from an anonymous structured questionnaire distributed to coeliac adult subjects. Data were recorded during the period from April to September 2018. Initially, on the basis of a literature review of the medical conditions potentially associated with coeliac disease, a specific questionnaire was developed to identify clinical systemic signs and symptoms, signs concerning the oral cavity (halitosis, reflux, oral lesions, caries, etc.), and the lifestyle of the enrolled patients. Then, the questionnaire was proposed to a sample of coeliac adult patients. These subjects were informed about this project through connection platforms (Facebook) and/or through advertisements in restaurants or supermarkets specializing in coeliac customers. A group of 237 patients participated and completed the anonymous questionnaire on-line via Google Forms.

The participants did not have to specify any demographic data (for example name, surname, or telephone number), as the questionnaire remained completely anonymous. They were invited to complete the questionnaire sincerely, and to give full importance to the project. Data management and analyses were organized to guarantee the participants’ full privacy.

### 2.1. The Questionnaire

First, useful information in order to understand the clinical history of the coeliac patient was gathered. Then, the questionnaire attempted to identify the main systemic diseases that affected the patients. The subjects were asked to select all items of interest in order to identify the pathologies potentially related to coeliac disease (for example, autoimmune thyroiditis, rheumatoid arthritis, type 1 diabetes, autoimmune hepatitis, osteoporosis, fertility disorders, dermatitis herpetiformis, selective IgA deficiency, Sjorgen syndrome, primary biliary cirrhosis, Crohn’s disease, Down’s syndrome, Ullrich-Turner syndrome, Williams syndrome, epilepsy). Then, a series of questions were asked in order to identify all the symptoms that may have characterized the patients’ clinical history. As coeliac disease is a multifactorial disease that can occur in different forms, it is important to identify correlations between symptoms; for this reason a series of questions concerned general symptoms (short stature, weight loss, puberty delay, asthenia, apathy, malaise, edema); gastrointestinal symptoms (dyspepsia, nausea, diarrhea, vomiting, constipation, abdominal distention, flatulence); neurological-psychiatric symptoms (peripheral neuropathy, ataxia, epilepsy, paresthesia, anxiety, depression, irritability); hematological problems (anemia, iron deficiency, folate deficiency, bleeding, ecchymosis); osteoarticular and muscular disorders (arthritis, osteoporosis, osteo-malacia, cramps, myopathy).Then, the main eating habits of the subjects were collected, followed by questions about their daily life.

A gluten-free diet is essential for the coeliac patient, but not all patients follow it with dedication. So some questions about gluten-free diet were included. In addition, it was requested if any other food had been removed from the patients’ diets for medical reasons: for example, lactose, sugars, red meats, white meats, sausages, or fat. Finally, a group of questions concerned oro-dental characteristics that may be congenital (glossitis, agenesis of teeth, etc.), or acquired, e.g., oral ulcer. Clinical data were requested before and after beginning an appropriate diet, in order to understand any changes in the acquired pathologies (bad breath, gastroesophageal reflux, xerostomia, humming, tension-type headache, joint clicks). In addition, questions about the home oral hygiene and prevention programs of the patient were addressed (for example, the frequency of dental appointments, the occurrence of recurring caries, and/or gingivitis signs and symptoms, dentin sensitivity, bleeding after brushing).

### 2.2. Statistical Analysis

Once a considerable number of responses were collected on time, the anonymous data were entered into an Excel data collection file, so that each response could be analyzed correctly. This file was then transferred to a statistical analysis program (SPSS—Hong Kong—Ltd., Rm 1804, 18/F, Westlands Road, Quarry Bay, Hong Kong, China). Data were dichotomized as presence or absence of a sign (or a symptom), or yes or no for a particular habit (concerning lifestyle). The results are shown as percentages of subjects answering yes or not, present or absent. Cross-tabulations and Odds Ratio (with confidence interval) are showed when statistically significant. The *p* value was set at 0.5%.

## 3. Results

237 individuals were recruited and participated to the project, answering the anonymous questionnaire. 100% of the subjects fully completed the questionnaire. Among them, 182 (76.7%) were female. 64 patients (27%) aged 15 to 24 years, 159 (67%) aged 25 to 55 and 14 (6%) aged 56 and over. 211 individuals (89%) reported adherence to a gluten-free diet, while 25 (10%) reported not adhering at all. One subject (0.42%) reported not following any diet.

Some differences in oral manifestations were observed between males and females. Regarding caries prevalence, for example, out of 182 (76.79%) coeliac females, 43 (18.14%) had caries, while out of 55 (23.21%) coeliac males, only 6 (12.24%) had caries. It can be observed that coeliac males predisposition to caries is about two times (OR: 0.40; CI: 0.16–0.99) lower than coeliac females. In addition, regarding dentin sensitivity, out of 182 (76.79%) coeliac females, 89 (37.55%) suffered from dentin sensitivity, whereas out of 55 (23.21%) coeliac males, only 14 (5.91%) suffered from dentin sensitivity. It can be observed that coeliac males are predisposed to suffer from dentin sensitivity almost 2. 5 times (OR: 0.36; CI: 0.18–0.70) less than females.

In addition, some oral manifestations resulted in significant correlation with the age of the subjects, as evidenced in Table 1. For example, the percentage of coeliac patients with gingivitis signs and symptoms increased significantly as the age group advanced. Similarly, the percentage of coeliac patients with dentin sensitivity increased significantly with increasing age.

In general, among coeliac patients with particular general health conditions correlated to coeliac disease (for example, illness, weight loss, short stature, puberty delay), the oral manifestations appeared evenly distributed (Table 2). But it can be noted that coeliac patients who didn’t manifest any particular general health condition (a sub-group of 45 coeliac subjects, 18.99% of the whole sample, described in Table 2) showed—for the most part (40 subjects, 88.89% of the sub-group)—no significant oral manifestation after gluten-free diet assumption (Table 2). In addition, 42 subjects out of 45 (93.33%) did not present halitosis. 39 subjects out of 45 (86.67%) did not suffer from nocturnal snoring (Table 2).

Some differences were observed between coeliac patients without gastrointestinal symptoms (a sub-group of 49 subjects) and those with these symptoms (a sub-group of 188 subjects) (Table 3). In the sub-group of 49 coeliac patients who did not report gastrointestinal symptoms, 40 subjects found no changes in their oral cavity after gluten-free therapy, whereas out of 188 patients with such problems, oral cavity changes were found by 77 (32.49%) patients. It can therefore be said that coeliac patients with associated gastrointestinal symptoms showed a risk of presenting oral manifestations about three times higher (OR: 3. 08; CI: 1.41–6.72) than those without them.

Some statistically significant differences were also observed between coeliac patients suffering from neuropsychiatric disorders (a sub-group of 79 subjects) and those without these disorders (a sub-group of 147 subjects) (Table 4). Out of the 79 patients suffering from psychiatric disorders, 38 subjects reported sporadic gingival bleeding, while 15 reported frequent bleeding. In the other cases, the bleeding was equally distributed.

In addition, some correlations were observed in the sub-group of coeliac patients reporting associated hematological problems (161 subjects), as 68 subjects out of 161 reported specific oral characteristics (Table 5). Among the 76 coeliac patients without hematological problems, 58 subjects found no changes in their oral cavity. Therefore, coeliac patients with hematological problems showed a risk of presenting changes in the oral cavity more than two times higher (OR: 2. 36; CI: 1.27–4.36) than those who did not have them. More specifically, among 76 subjects without hematological problems, 66 patients did not show any enamel defects, while among 161 patients with hematological problems, 43 subjects reported dental enamel defects. Therefore, coeliac patients with hematological problems can be said to have a 2.5 times higher risk of showing enamel defects (OR: 2.67; CI: 1.25–5.82) than patients who do not have them. Also, coeliac patients with hematological problems have an almost four times higher risk of presenting xerostomia (OR: 3. 80; CI 1.42–10.16) than those without hematological problems (out of 76 coeliac patients without hematological problems, 71 did not have xerostomia, while among 161 patients with hematological problems, 34 subjects had xerostomia). The same was found for dentin sensitivity. It can be observed that coeliac patients with hematological problems have a risk of experiencing dental sensitivity more than two times higher (OR: 2. 28; CI: 1.28–4.06) than those who do not.

In addition, some statistically significant correlations were observed in the sub-group of coeliac patients reporting muscular disorders (a sub-group of 60 subjects) (Table 6). Among 177 coeliac patients without muscular disorders, 121 reported no changes in their oral cavity following gluten-free therapy, while out of 60 coeliac patients with such problems, oral changes after diet were reported by 30 subjects. Therefore, it can be stated that coeliac patients with muscular disorders have a risk of presenting changes at the level of the oral cavity after the adoption of a gluten-free diet that is twice as high (OR:2.16; CI:1.19–3.93) compared to those who do not have these problems. In addition, coeliac patients with concurrent muscular disorders showed a risk (OR: 1.98; CI 1.07–3.64) of suffering from TMJ clicks twice as high than those who do not suffer from muscular disorders. Finally, it can be said that coeliac patients with muscular disorders have a risk of presenting dentin sensitivity approximately 2.5 (OR: 2.46; CI: 1. 35–4. 48) times higher than those who do not suffer from muscle disorders.

Other correlations were observed between the total and partial gluten-free diet (Table 7). The number of patients with gingival bleeding and partial gluten-free diet was 117 out of 211, while the number of patients on a totally gluten-free diet and gingival bleeding was 11 out of 25. In addition, the number of patients with nocturnal snoring among subjects with a partial gluten-free diet was 35 out of 211, whereas among patients with a totally gluten-free diet, nocturnal snoring resulted in 2 out of 25 patients.

Finally, some correlations were observed with the removal of particular foods (Table 8). Out of 123 patients who did not give up any food, 92 subjects did not report any changes in the oral cavity with the introduction of the gluten-free diet. But out of only five patients who did not eat meat, four reported changes in their oral cavity with the introduction of the gluten-free diet. Out of 123 (51.90%) patients who did not give up any food, only three suffered from glossitis. But out of five (2.11%) patients who did not eat meat, two subjects suffered from glossitis. In addition, out of 123 patients who did not give up any food, only 14 reported the disappearance of aphthous lesions after the introduction of the gluten-free diet. Of the eight patients who do not eat fat foods, three reported the total disappearance of mouth ulcers with the introduction of a gluten-free diet.

## 4. Discussion

In this study, 237 individuals suffering from coeliac disease were recruited through connection platforms such as Facebook and in restaurants and supermarkets specializing in coeliac clients. According to the Annual Report to the Parliament on Coeliac Disease, at the end of 2016 there were 198,427 coeliac patients in Italy, instead of the approximately 600,000 expected. Coeliac females (138,902 in 2016) were more than twice the number of males (59,525).

From the present findings, some differences in oral manifestations were observed between males and females. Regarding caries prevalence, for example, coeliac females were almost twice as susceptible to caries than men. This result is not clearly confirmed in the literature as far as the healthy population is concerned, but there are data (relating to the non-coeliac population) that suggest that there could be such a difference in the predisposition to caries between the two genders. For example, the study by Galvao-Moreira et al. [7] shows that female salivary pH is more acidic than male pH, and the same was suggested by the study by Eliasson et al. [8] in which lower rates of buccal and labial salivation were found in females, as well as lower levels of IgA.

In addition, some statistically significant correlations were found in relation to the age of the subjects. For example, with progressing age, the prevalence of dentin sensitivity and TMJ clicks increased, with a peak prevalence in subjects aged between 45 and 64. The analysis of the data on the prevalence of TMJ clicks in the various age groups is confirmed by previous literature [9,10], and this ensures the reliability of the data collected.

In the literature it has been demonstrated that coeliac disease presents a heterogeneity in the manifestation of symptoms, and therefore there are cases with manifestations of “minor” entity, and clinical cases in which the symptoms are more evident and heterogeneous. From the present study, it appears evident that clinical cases with a more evident manifestation of general coeliac characteristics (i.e., those cases in which the coeliac patients also present general related characteristics) are the same with a greater symptomatology at the level of the oral cavity as well. This aspect has been observed, for example, for related pathologies such as hematological systemic disorders, for symptoms of muscle disorders, and for neuro-psychiatric symptoms.

From the present survey, examples of oral cavity interest in coeliac patients with related pathologies are the observed correlation between tension-type headache and dentin sensitivity, and between general symptoms in coeliac patients (such as gastroesophageal reflux) and halitosis, that are often also associated in non-coeliac subjects [11,12]. Dentin sensitivity was also found to be associated with coexistence of other related diseases in coeliac patients, such as xerostomia. This result is most likely to be related to the fact that coeliac sufferers often present enamel defects [13,14,15] which could lead to dentin sensitivity. The literature partly seems to confirm these associations. For example, with regard to the association between xerostomia and coeliac disease, the study by van Gils et al. [16] reports the evaluation of 740 patients with coeliac disease and 270 control subjects, showing that oral health problems are more commonly experienced in adult patients with coeliac disease than in the comparison group. Regarding the association between halitosis and coeliac disease, in the literature the study by Tsai et al. [6], based on a sample of children, states that there is a correlation. Finally, regarding the association between tension-type headache and coeliac disease, the literature seems to confirm the present observation. The study by Zis et al. [4] states that the average aggregate prevalence of tension-type headache among coeliac patients is 26% in adult populations. The study concludes by inviting patients with headache of unknown origin to be screened for coeliac disease, as these patients could benefit from a gluten-free diet.

According to the present findings following a gluten-free diet appears crucial to managing oral diseases associated to coeliac pathology. In fact, gingival bleeding levels increase with a high systemic inflammatory rate (i.e., the detection of a whole series of inflammatory symptoms related to coeliac disease), in coeliac patients who do not follow a gluten-free diet carefully. In addition to gingivitis management with chlorhexidine [17], a general program of prevention in these subjects should also be recommended. A previous NHANES (National Health and Nutrition Examination Survey) study, apparently in disagreement with the present findings, showed the absence of association of coeliac disease with periodontal disease and the absence of difference between subjects with diagnosed and undiagnosed coeliac disease, but the sample size of the coeliac disease group was low and the severity of the general and oral manifestations of the disease was not considered as a possible confounding factor [18].

Another example of the potential role of the gluten-free diet was observed on nocturnal snoring, a condition which is now increasingly managed in the dental field, as the data of the present study reveal that a worsening in nocturnal snoring is observed when the coeliac patient does not strictly follow the correct diet.

Even if previous studies have also reported the manifestations of coeliac disease on oral health [13,14], to the authors’ knowledge this is the first study that evidences the potential importance of following a strictly gluten-free diet in controlling gingival bleeding levels and nocturnal snoring in patients with coeliac disease. Thus, clinical studies should be encouraged to confirm these results and estimate the impact of the gluten-free diet on periodontal indices and sleep-related breathing disorders of subjects affected by coeliac disease.

In general, it can be said that the results of the present study showed that an adult coeliac patient with associated systemic diseases could also present a significant prevalence of diseases and symptoms at the level of the oral cavity. Patients with fewer “systemic” symptoms, on the other hand, show more modest oral symptoms. The present data suggest that it is essential to monitor frequently, over time, the general health of the coeliac patient, as well as his/her oral health, as the presence of symptoms of inflammation at the level of the oral cavity is often associated with systemic symptoms [19,20,21] that can be also related to coeliac disease. Therefore, the prevention of inflammatory diseases in the oral cavity inevitably includes a prevention program that invests in the general health of the patient and dentists are also called upon to take part because of the particular role they play in care of the oral cavity.

Nowadays, it is increasingly clear that the figure of the dentist not only has the task of monitoring oral diseases but is often identified as an educator regarding a healthy lifestyle and proper nutrition.

## 5. Conclusions

The results of the present study suggest that the dentist should implement a specific clinical protocol for coeliac patients, due to the peculiar and heterogeneous clinical situation that they may present. This protocol should include frequent follow-ups with monitoring of “general” health, in addition to oral health, including several recommendations for compliance with the gluten-free diet. In fact, following a gluten-free diet could be important to control gingival bleeding levels and to manage oral symptoms associated with coeliac disease.

In addition to monitoring the appearance of specific symptoms and signs in the mouth, the dentist should encourage the patient to perform other general health checks as well.

## Figures and Tables

**Table 1:** Age range of subjects included in the study, and oral manifestations. Statistically significant differences.

Oral Manifestations		Age Range			*p* Value
		0–25 Years (64 Subjects)	26–55 Years (159 Subjects)	>55 Years (14 Subjects)	
Modifications in the oral manifestations after gluten-free diet	NO	49 (76.56%)	97 (61.01%)	5 (35.71%)	0.007
	YES	15 (23.44%)	62 (38.99%)	9 (64.29%)	
Gingivitis signs and symptoms	NO	51 (100%)	122 (89.71%)	10 (71.43%)	0.003
	YES	0 (0%)	14 (10.29%)	4 (28.57%)	
Dentin sensitivity	NO	47 (73.44%)	81 (50.94%)	6 (42.86%)	0.005
	YES	17 (26.56%)	78 (49.06%)	8 (57.14%)	

**Table 2:** Associations between general health symptoms and oral manifestations.

Oral Manifestations		General Health Particular Condition Correlated To Coeliac Diases						*p* Value
		None (45 Subjects)	Illness (85 Subjects)	Puberty Delay (2 Subjects)	Weight Loss (19 Subjects)	Short Stature (9 Subjects)	Combined Symptoms (77 Subjects)	
Modifications in the oral cavity after gluten-free diet	NO	40 (88.89%)	51 (60%)	1 (50%)	12 (63.16%)	4 (44.44%)	43 (55.84%)	0.006
	YES	5 (11.11%)	34 (40%)	1 (50%)	7 (36.84%)	5 (55.56%)	34 (44.16%)	
Halitosis	NO	42 (93.33%)	67 (78.82%)	0 (0%)	18 (94.74%)	6 (88.67%)	63 (81.82%)	0.004
	YES	3 (6.67%)	18 (21.18%)	2 (100%)	1 (5.26%)	3 (33.33%)	14 (18.18%)	
Gastroesophageal reflux	NO	41 (91.11%)	56 (65.88%)	1 (50%)	13 (68.42%)	8 (88.89%)	45 (58.44%)	0.005
	YES	4 (8.86%)	29 (34.12%)	1 (50%)	6 (31.58%)	1 (11.11%)	32 (41.56%)	
Nocturnal snoring	NO	39 (86.67%)	64 (75.29%)	1 (50%)	16 (84.21%)	9 (100%)	70 (90.91%)	0.048
	YES	6 (13.33%)	21 (24.71%)	1 (50%)	3 (15.79%)	0 (0%)	7 (9.09%)	

**Table 3:** Associations between gastrointestinal symptoms and oral manifestations.

Oral Manifestations		Gastrointestinal Symptoms				
		NO (49 Subjects)	YES (188 Subjects)	OR ^1^	CI ^2^	*p* Value
Changes in the oral cavity after gluten-free diet	NO	40 (81.63%)	111 (59.04%)	3.08	1.41–6.72	0.003
	YES	9 (18.37%)	77 (40.96%)			

^1^ Confidence Interval; ^2^ Odds Ratio.

**Table 4:** Associations between neuropsychiatric disorders and oral manifestations.

Oral Manifestations		Neuropshychiatric Disorders				*p* Value
		No (147 Subjects)	Psychiatric (79 Subjects)	Neurological (5 Subjects)	Combined (6 Subjects)	
Modifications in the oral cavity after gluten-free diet	NO	111 (75.51%)	38 (48.10%)	2 (40%)	0 (0%)	<0.001
	YES	36 (24.29%)	41 (51.60%)	3 (60%)	6 (100%)	
Gastroesophageal reflux	NO	114 (77.55%)	45 (56.96%)	45 (56.96%)	2 (33.33%)	
	YES	33 (22.45%)	34 (43.04%)	34 (43.04%)	4 (66.67%)	
Enamel defects	NO	120 (81.63%)	59 (75.64%)	2 (40%)	3 (50%)	0.04
	YES	27 (18.37%)	19 (24.36%)	3 (60%)	3 (50%)	
Gingival bleeding	NO	76 (51.70%)	26 (32.91%)	3 (60%)	3 (50%)	0.003
	SOMETIMES	64 (43.54%)	38 (48.10%)	2 (40%)	1 (16.67%)	
	YES	7 (4.76%)	15 (18.99%)	0 (0%)	2 (33.33%)	
Aphthous stomatitis	NO	110 (74.83%)	45 (56.96%)	1 (20%)	4 (66.67%)	<0.001
	PRE-DIET	19 (12.93%)	12 (15.19%)	4 (80%)	1 (16.67%)	
	YES	18 (12.24%)	22 (27.85%)	0 (0%)	1 (16.67%)	
Xerostomia	NO	129 (87.76%)	59 (74.68%)	5 (100%)	5 (83.33%)	0.06
	YES	18 (12.24%)	20 (25.32%)	0 (0%)	1 (16.67%)	
Articular clicks	NO	110 (74.83%)	47 (59.49%)	5 (100%)	3 (50%)	0.029
	YES	37 (25.17%)	32 (40.51%)	0 (0%)	3 (50%)	
Tension-type headache	NO	96 (65.31%)	40 (50.63%)	1 (20%)	3 (50%)	0.045
	YES	51 (34.69%)	39 (49.37%)	4 (80%)	3 (50%)	
Dentin sensitivity	NO	95 (64.63%)	34 (43.04%)	3 (60%)	2 (33.33%)	0.011
	YES	52 (35.37%)	45 (56.96%)	2 (40%)	4 (66.67%)	

**Table 5:** Associations between hematological disorders and oral manifestations.

Oral Manifestations		Hematological Disorders				
		NO (76 Subjects)	YES (161 Subjects)	OR ^1^	CI ^2^	*p* Value
Modifications in the oral cavity after gluten free diet	NO	58 (76.32%)	93 (57.76%)	2.36	1.27–4.36	0.006
	YES	18 (23.68%)	68 (42.24%)			
Enamel defects	NO	66 (88.00%)	118 (73.29%)	2.67	1.23–5.82	0.011
	YES	9 (12.00%)	43 (26.71%)			
Xerostomia	NO	71 (93.42%)	127 (78.88%)	3.8	1.42–10.16	0.005
	YES	5 (6.58%)	34 (21.12%)			
Dentin sensitivity	NO	53 (69.74%)	81 (50.31%)	2.28	1.28–4.06	0.005
	YES	23 (30.26%)	80 (49.69%)			

^1^ Confidence Interval; ^2^ Odds Ratio.

**Table 6:** Associations between muscular disorders and oral manifestations.

Oral Manifestations		Muscular Disorders				
		NO (177 Subjects)	YES (60 Subjects)	OR ^1^	CI ^2^	*p* Value
Modifications in the oral cavity after gluten free diet	NO	121 (68.36%)	30 (50.00%)	2.16	1.19–3.93	0.011
	YES	56 (31.64%)	30 (50.00%)			
Gastroesophageal reflux	NO	132 (74.58%)	32 (53.33%)	2.57	1.40–4.72	0.002
	YES	45 (25.42%)	28 (46.67%)			
Xerostomia	NO	156 (88.14%)	42 (70.00%)	3.18	1.56–6.51	0.001
	YES	21 (11.86%)	18 (30.00%)			
Gingivitis signs and symptoms	NO	143 (93.46%)	40 (83.33%)	2.86	1.06–7.72	0.032
	YES	10 (6.54%)	8 (16.67%)			
Articular clicks	NO	130 (73.45%)	35 (58.33%)	1.98	1.07–3.64	0.028
	YES	47 (26.55%)	25 (41.67%)			
Dentin sensitivity	NO	110 (62.15%)	24 (40.00%)	2.46	1.35–4.48	0.003
	YES	67 (37.85%)	36 (60.00%)			

^1^ Confidence Interval; ^2^ Odds Ratio.

**Table 7:** Associations between partial/total gluten free diet and oral manifestations.

Oral Manifestations		Diet			*p* Value
		None (1 Subject)	Total Gluten Free Diet (211 Subjects)	Partial Gluten Free Diet (25 Subjects)	
Bleeding	NO	0 (0%)	94 (44.55%)	14 (56%)	0.039
	SOMETIMES	0 (0%)	96 (45.50%)	9 (36%)	
	YES	1 (100%)	21 (9.95%)	2 (8%)	
Nocturnal snoring	NO	0 (0%)	176 (83.41%)	23 (92%)	0.039
	YES	1 (100%)	35 (16.59%)	2 (8%)	

**Table 8:** Associations between foods consumption and oral manifestations.

Oral Manifestations		Other Food						*p* Value
		None (123 Subjects)	Lactose (45 Subjects)	Sugar (11 Subjects)	Meat (5 Subjects)	Fat (8 Subjects)	Combined (45 Subjects)	
Modifications in the oral cavity after gluten-free diet	NO	92 (74.80%)	23 (51.11%)	8 (72.73%)	1 (20%)	4 (50%)	23 (51.11%)	0.003
	YES	31 (25.20%)	22 (48.89%)	3 (27.27%)	4 (80%)	4 (50%)	22 (48.89%)	
Gastroesophageal reflux	NO	95 (77.24%)	27 (60%)	7 (63.64%)	0 (0%)	8 (100%)	27 (60%)	<0.001
	YES	28 (22.76%)	18 (40%)	4 (36.36%)	5 (100%)	0 (0%)	18 (40%)	
Symptoms of Glossitis	NO	120 (97.56%)	43 (95.56%)	10 (90.91%)	3 (60%)	7 (87.50%)	42 (93.33%)	0.007
	YES	3 (2.44%)	2 (4.44%)	1 (9.09%)	2 (40%)	1 (12.50%)	3 (6.67%)	
Aphthous lesions	NO	92 (74.80%)	23 (51.11%)	9 (81.82%)	1 (20%)	5 (62.50%)	30 (66.67%)	0.002
	PRE-DIET	14 (11.38%)	11 (24.44%)	1 (9.09%)	0 (0%)	3 (37.50%)	7 (15.56%)	
	YES	17 (13.82%)	11 (24.44%)	1 (9.09%)	4 (80%)	0 (0%)	8 (17.78%)	
